# Evaluation of (CNT@CIP)-Embedded Magneto-Resistive Sensor Based on Carbon Nanotube and Carbonyl Iron Powder Polymer Composites

**DOI:** 10.3390/polym14030542

**Published:** 2022-01-28

**Authors:** Daeik Jang, Jae-Eun Park, Young-Keun Kim

**Affiliations:** 1 Department of Civil and Environmental Engineering, KAIST, 291 Daehak-ro, Yuseong-gu, Daejoen 34141, Korea; svs2002@kaist.ac.kr; 2 Department of Mechanical and Control Engineering, Handong Global University, 558 Handong-ro, Pohang 37554, Korea; jepark@handong.edu

**Keywords:** carbon nanotubes (CNTs), carbonyl iron powder (CIP), magneto-resistive, sensors

## Abstract

The conductive polymeric composites incorporating carbon nanotube (CNT) and carbonyl iron powder (CIP) have attracted much attention for various sensor applications. In this paper, a comprehensive study of the magneto-sensing property of a CNT-CIP embedded polymer composite is conducted to implement the composite as magneto-sensors. Thus, this study experimentally investigated the magneto-sensing performances of CNT-doped polymeric composites with the addition of CIP in terms of electrical conductivity, sensitivity, repeatability, and response time. First, the CNT-CIP clusters were manufactured and their interactions were analyzed with the zeta potential measurement and SEM observation. Then, the CNT-CIP clusters were embedded into the polymeric composites for the magneto-sensing evaluations. Experiments showed that the CNT contents in the range of percolation threshold (i.e., 0.5% and 0.75%) are optimal values for sensor applications. The addition of CNT 0.5% and 0.75% resulted in a high sensitivity of 7% and a faster response time within 400 ms. Experiment evaluation confirmed a high potential of implementing CNT-CIP composite as magneto-sensors.

## 1. Introduction

Expeditious advancements of conductive polymeric composites (CPCs) have been gaining attention due to their high applicability, such as multi-functional sensors [1,2,3], electrical-heating composites [4,5,6], electromagnetic interference shielding composites [7,8,9], and other electronic devices [10,11]. Various kinds of conductive fillers including carbon-based fillers such as graphene, and carbon nanotubes and magnetic powder have been used to fabricate the CPCs due to their high electrical conductivity [11,12,13]. Among the conductive fillers, carbon nanotube (CNTs) have been popular conductive fillers due to their remarkable electrical and mechanical properties and high compatibility with polymer matrix. In addition, many researchers have attempted to use two types of conductive fillers to improve their mechanical, electrical, and sensing properties [14]. Among various conductive fillers, the carbonyl iron powder (CIP) is well known for the high magnetization property. The CIP fillers in a polymeric composite respond to the magnetic field and can improve both the electrical conductivity and sensing capability of CNT-doped polymeric composites [15,16]. Most of the related literature focused on fabricating CIP polymeric composites and investigating the magneto-resistive sensing performances [17,18]; however, there is a lack of a comprehensive study for observing magneto-resistive sensing performances of sensitivity, response time, and repeatability. Furthermore, the investigations on electrostatic interaction that form the CIP-CNT clusters and the study of CNT effects on the magneto-sensing performances have not been reported to date, to the best of the authors’ knowledge. Therefore, this paper is focused on investigating the (1) mechanisms of forming CIP-CNT clusters, (2) effects of CNT inclusion on the polymer sensing performances under magneto-induced conditions, and (3) performance of the magneto-resistive sensing of the CIP-CNT polymer.

## 2. Experimental Section

### 2.1. Sample Preparation

**Figure 1:**
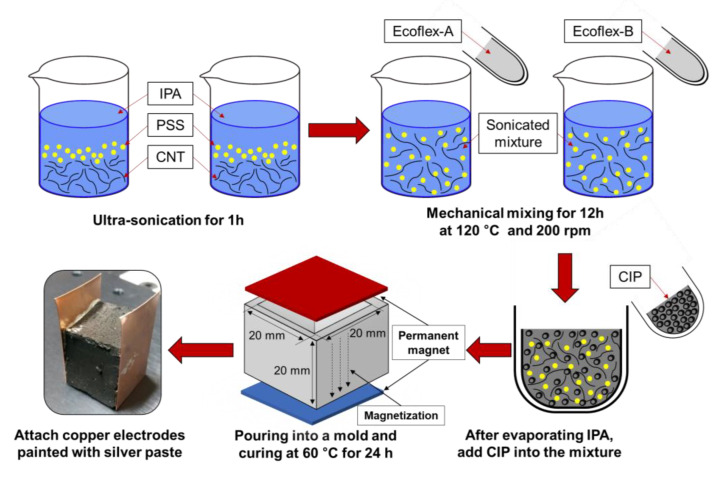
The fabrication process of the samples for magneto-sensor application.

In the present study, Ecoflex^®^ 0010 of poly-butylene adipate-co-terphthalate (PBAT) is utilized as the polymeric matrix. It is a flexible polymer matrix, widely used in related research [5,18], and the detailed chemical specification Ecoflex^®^ 0010 is explained in the study by Wei et al. [19]. Carbonyl iron powder (CIP) and multi-walled carbon nanotube (CNTs) were used as conductive fillers to fabricate the polymeric composites. Here, CNTs were included in the composites for ensuring favorable electrical conductivity; thus, the target electrical resistance of the fabricated samples was decided as less than 1 kΩ. To obtain the proper CNTs contents, four different CNTs (0.5, 0.75, 1.0, and 2.0 wt.%) were chosen to fabricate the samples. The detailed specifications of the utilized CNTs and CIP were summarized in Table 1. The polysodium4-styrenesulfonate (PSS) was utilized to disperse the CNT bundles in the polymeric matrix [20]. The fabrication process of the samples is described in Figure 1. CNTs and identical amounts of PSS were added to a beaker with 100 mL of volatile solvent (Isopropyl-alcohol, IPA), and the mixtures were hand-mixed for 1 min. Afterward, they were sonicated for an additional 1 h via a bath-typed ultra-sonicator (40 kHz; 200 W). Ecoflex^®^ was then added to the sonicated mixtures, and they were heated for 12 h at 120 °C with stirred at 200 rpm to both improve the dispersion of CNTs and evaporate the IPA solvent in the Ecoflex^®^ matrix. After 12 h, CIP 50% by polymer weight was added to the mixtures, and they were hand-mixed for an additional 3 min. The mixtures were cured in a 20 × 20 × 20 mm^3^ mold at an oven with a temperature of 60 °C for 24 h (Figure 1). During the curing, the samples were located under the magnetic field to form the CIP-based chain structures in the samples as described in the fabrication process of samples in the previous study [18,20].

### 2.2. Experiment Methods

A zeta potential analyzer (ELSZ-2000, Otsukael, Otsuka Electronics, Osaka, Japan) was used to measure the potential differences of the used materials in IPA solvent. A micro-structural image of the sample was taken through field emission scanning electron microscopy (FE-SEM, Hitachi S4800, Hitachi, Ltd., Chiyoda City, Tokyo, Japan), and they were discussed with the formation of electrical conductivity networks and the overall magneto-resistive sensing test results. The electrical resistance of the samples was measured using a portable digital multi-meter with a two-probe method, and they were converted to the electrical resistivity. A total of three samples in each mix proportion were chosen to calculate the average values. Input voltage (1 V) was applied to the samples and their electrical resistance variations were measured to observe the electrical stability of the samples under the static conditions for 4 h. The magneto-resistive sensing performances of the samples were evaluated with and without a magnetic field. Magnetic field levels (0 and 200 mT) were applied to the samples for 30 s, respectively, and the corresponding electrical resistances of the samples were measured simultaneously. The identical process was repeated for 3 cycles for every sample. The magneto-sensing performances of the samples including sensitivity and response time were evaluated in terms of the resistance change over time.

## 3. Results and Discussion

### 3.1. Development of CIP@CNT Clusters

**Figure 2:**
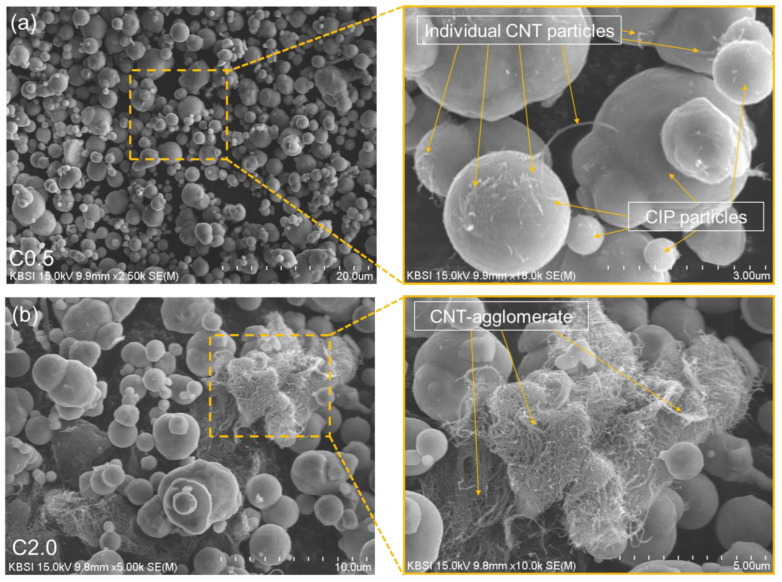
SEM images of CNT and CIP-based conductive networks in the samples with (**a**) CNT 0.5% and (**b**) CNT 2.0%.

First, the CIP-based clusters coated with CNT were developed. Then, their formation was investigated using the zeta potential analysis and SEM observation. In Table 2, the relative zeta potential values of CNTs and CIP powders were 3.33 eV and −29.73 eV, respectively. In zeta potential analysis, the particles charged oppositely mean that they can be hybridized based on the electrostatic interaction [5]. The hybridized of CNTs and CIP (i.e., CIP@CNT clusters) can be verified by the relative zeta potential values of CNTs and CIP in IPA solvent (i.e., −4.17 eV). This hypothesis can be also supported by the SEM images as shown in Figure 2. As expected, CIP was wrapped with the individual CNT particles due to the electrostatic interaction, which showed in close agreement with the zeta potential test results (Table 2). In SEM images, it can be found that the addition of both CNTs and CIP into the polymeric composites can form the CIP@CNT clusters. The clusters can change their original position as the magnetic field is applied due to the movements of CIP, leading to a change in the distances between the CNT particles. Accordingly, this micro-structural analysis can verify the development of CIP@CNT clusters and explain the principles of magneto-sensing.

### 3.2. Electrical Characteristics

**Figure 3:**
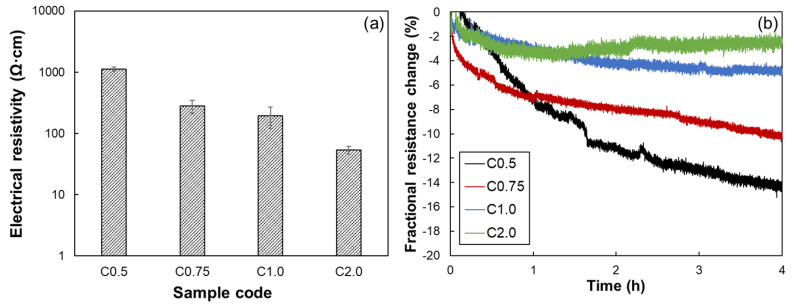
(**a**) Electrical resistivity and (**b**) tunneling-induced electrical stability of samples.

The electrical resistivity values of the samples are shown in Figure 3. The electrical resistivity of the samples decreased from 1.13 kΩ·cm to 54 Ω·cm as the incorporated CNTs content increased from 0.5 to 2.0%, respectively. According to these results, it can be said that the dramatic reduction in electrical resistivity was found in the incorporated CNTs contents range from 0.5 to 0.75%. This range is called a percolation threshold where the denser conductive networks start to form, decreasing the electrical resistivity dramatically. The percolation threshold range found in the present study is similar to the previous studies, indicating the well-dispersion of the CNTs in the samples (Figure 3a). The electrical stability of the samples for 4 h is illustrated in Figure 3b. The fractional resistance change of C0.5 sample after 4 h was approximately −14.5%, while that of C0.75, C1.0, and C2.0 samples were −10.15, −4.86, and −2.20%, respectively. This result can be explained by the tunneling effects which are commonly known as the nature of CNTs. Due to the tunneling effect, the electrons can skip over the insulating polymer matrix and move easily to the adjacent CNT particles when the electrical current has applied to the composites [21]. Thus, the electrical resistance between the CNT particles is decreased over time with increasing the electrical conductivity of the composites, as seen in Figure 3b. In particular, it can be observed that the tunneling effect decreased as the incorporated CNTs content increased (Figure 3b). As the incorporated CNT content increases, the adjacent CNT particles draw closer. The shortened distance between CNT particles reduces the tunneling effect because the tunneling effect is inversely proportional to the distance between CNT particles, showing close agreement with the previous studies [5,21].

### 3.3. Magneto-Resistive Sensing Performances

**Figure 4:**
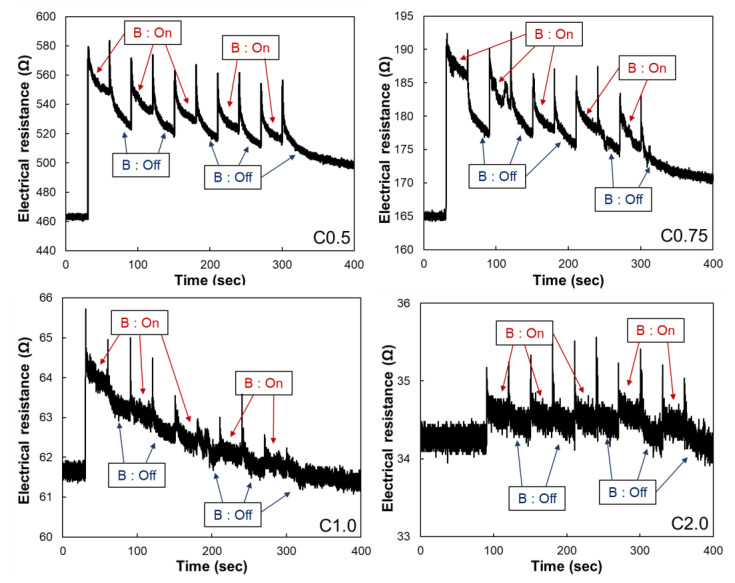
Magneto-resistances of the samples with various CNTs contents.

The magneto-resistive sensing performances of the samples under the different levels of the applied magnetic field are displayed in Figure 4. The electrical resistance increases as the magnetic field are applied to the samples regardless of the embedded CNT contents. This phenomenon can be explained by the re-positions of CNTs and CIP-based conductive networks. The CIP is wrapped with individual CNT particles, forming clusters composed of CNTs and CIP, and the clusters respond to the applied magnetic field. Thus, the distances of the individual CNT particles increase, and it leads to the increase in electrical resistance (Figure 4). However, the degree of fractional change in electrical resistance was significantly different in the samples according to the embedded CNT contents. The C0.5 and C0.75 samples showed approximately 8.3% and 5.8% of fractional electrical change, respectively, while those of C1.0 and C2.0 samples were less than 2%. This result is deduced from the percolation threshold observed in Figure 3. If the CNT contents are in the percolation threshold (C0.5 and C0.75), the small change in distances between CNT particles can lead to a high change in the electrical resistance. In addition, the change of distances between CNT particles caused by the applied magnetic field cannot affect the electrical resistance significantly. It is due to the high amount of CNT particles in C1.0 and C2.0 samples (1.0 and 2.0%) already forming the dense electrical conductive networks. In this regard, we conclude that the samples with CNT contents in the percolation threshold range are proper for magneto-sensors; thus, the C0.5 and C0.75 samples were chosen to use in further tests.

### 3.4. Sensitivity and Repeatability

**Figure 5:**
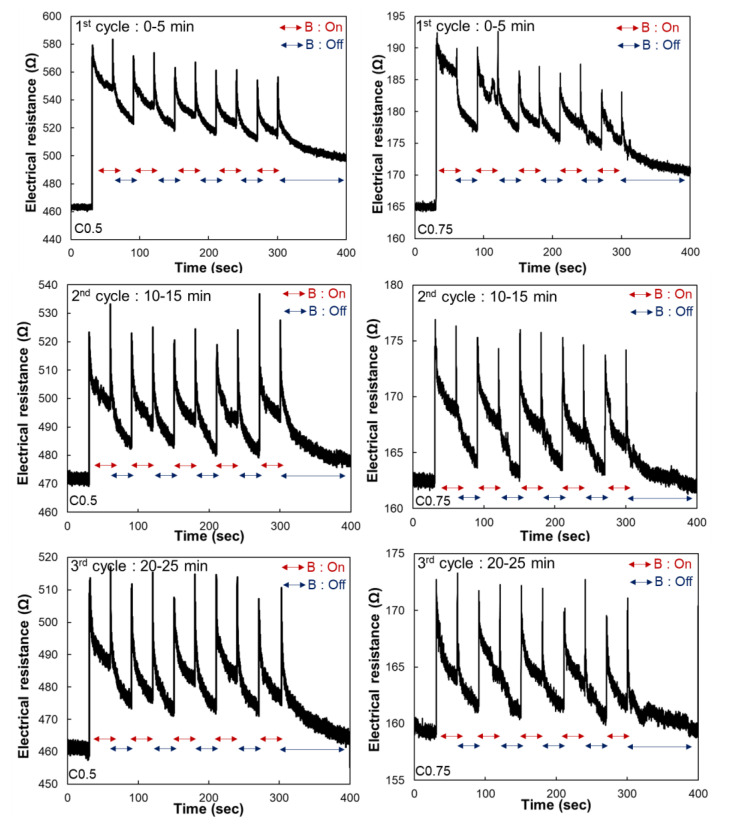
Magneto-resistances of the C0.5 and C0.75 samples under different levels of magnetic field.

**Figure 6:**
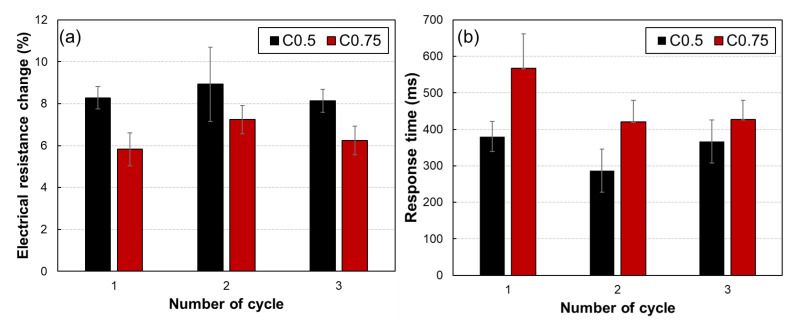
(**a**) Magneto-sensing sensitivity and (**b**) response time of C0.5 and 0.75 samples.

The process as described in Figure 4 was repeated for three cycles to C0.5 and C0.75 samples to investigate the sensitivity and repeatability, and the results are shown in Figure 5. The initial electrical resistances of the samples decreased with an increase in the cycles, regardless of the CNT contents, which can be explained by the tunneling effect as observed in Figure 3. Nonetheless, notable changes in electrical resistance were observed as the magnetic field was applied to both samples during the cyclic test, indicating a possibility of using them as magneto-sensors (Figure 5). Then, the magneto-sensing sensitivity and response time were investigated in Figure 6. As observed in Figure 6, the average reduction in electrical resistance of C0.5 sample caused by the applied magnetic field was approximately 42.9 Ω, 43 Ω, and 38 Ω in the first to the third cycle, respectively, and can be converted to 8.3%, 8.9%, and 8.1% of magneto-sensitivity, respectively. For the C0.75 sample, the variation in electrical resistance caused by magnetic field stimulus change was approximately 10 Ω to 11 Ω, indicating 5.8%, 7.2%, and 6.2% of magneto-sensitivity, respectively. Even the absolute value of electrical resistance changes over iterations, the magneto-sensing sensitivity is stable at approximately 8% and 6% for the C0.5 and C0.75 samples, respectively. In addition, the response time is calculated by observing the time interval between the instant the magnetic field is applied and the instant resistance peak is reached, as shown in Figure 6b. The response time of C0.5 and C0.75 samples exhibited about 350 ms and 450 ms, respectively. The fabricated samples showed similar values of response time with the values observed in previous studies that showed the response time of Ecoflex^®^-based composites around 332 ms due to the ultra-high viscoelastic properties [22,23].

## 4. Conclusions

In the present study, the experimental investigations about the electrical characteristics and magneto-resistive sensing performances of CIP@CNT clusters-embedded polymeric composites for sensor applications were evaluated. First, the CIP coated with CNT (CIP@CNT) clusters were fabricated, and their formation was investigated with the zeta potential measurement and SEM images. In observation of electrical characterization, the effects of included CNT contents on the electrical conductivity of samples were observed, and the percolation threshold (0.5% to 0.75%) was found. Through the magneto-resistive sensing test, the samples with CNT contents in the percolation threshold showed higher sensitivity, i.e., approximately 7%, and faster response within 400 ms compared to that found in the previous studies. Thus, the present experimental results exhibited that CIP@CNT clusters-embedded polymeric composites have potential applications as magneto-sensors. For the further work, the optimization of polymer matrix selection, proportions of CIP and CNT, and addition of other fillers are recommended to be conducted to maximize the overall magneto-resistive sensing performance. After the aforementioned research, the optimal magneto-resistive sensors based on the CIP@CNT-embedded polymeric composites are expected to be manufactured for practical and commercial applications.

## Figures and Tables

**Table 1:** Mix proportions of the specimens fabricated in the present study (wt.%).

Designation	PDMS		CNT	PSS	CIP
	Base	Curing Agent			
C0.5	100	10	0.5	0.5	30
C0.75	100	10	0.75	0.75	
C1.0	100	10	1.0	1.0	
C2.0	100	10	2.0	2.0	

**Table 2:** Zeta potential test results of the CNTs and CIP in IPA solvent.

Zeta Potential (mV)	IPA Solvent	CNTs in IPA	CIP in IPA	CNTs and CIP in IPA
Absolute value	−15.19	−11.86	−44.92	−19.36
Relative value	0	3.33	−29.73	−4.17

## Data Availability

The data presented in this study are available on request from the corresponding author.
